# Foliar application of zinc sulphate and zinc EDTA to wheat leaves: differences in mobility, distribution, and speciation

**DOI:** 10.1093/jxb/ery236

**Published:** 2018-06-21

**Authors:** Casey L Doolette, Thea L Read, Cui Li, Kirk G Scheckel, Erica Donner, Peter M Kopittke, Jan K Schjoerring, Enzo Lombi

**Affiliations:** 1 University of South Australia, Future Industries Institute, Mawson Lakes, South Australia, Australia; 2 The University of Queensland, School of Agriculture and Food Sciences, St. Lucia, Queensland, Australia; 3 U.S. Environmental Protection Agency, Office of Research and Development, Cincinnati, OH, USA; 4 Department of Plant and Environmental Sciences, Faculty of Science, University of Copenhagen, Frederiksberg C, Denmark

**Keywords:** Biofortification, chelated zinc, foliar fertilizer, plant nutrition, wheat, XANES, XFM, zinc, ZnEDTA

## Abstract

Foliar application of zinc (Zn) to crops is an effective way to increase the grain concentration of Zn. However, the development of more efficient foliar Zn fertilizers is limited by a lack of knowledge regarding the distribution, mobility, and speciation of Zn in leaves once it is taken up by the plant. We performed an experiment using radiolabelled Zn (^65^Zn), and *in situ* time-resolved elemental imaging using synchrotron X-ray fluorescence microscopy (XFM), to investigate the behaviour of two commonly used Zn foliar fertilizers (Zn sulphate and ZnEDTA) in wheat (*Triticum aestivum*) leaves. Both experiments showed that Zn had limited mobility in leaves, moving <25 mm from the application point after 24 h. Although limited, the translocation of Zn occurred quickly for both treatments; moving more between 3 h and 12 h after application than between 12 h and 24 h. Speciation analysis using synchrotron-based X-ray absorption near-edge structure (XANES) showed that ZnEDTA was in fact taken up in chelated form and not as ionic Zn (Zn^2+^). The XANES data also showed that Zn, from both treatments, was then complexed by ligands in the leaf (e.g. phytate and citrate), potentially in response to localized Zn toxicity. The results of the present study provide important insights into the behaviour of commonly used foliar-applied Zn fertilizers, and can be used to optimize current fertilization strategies and contribute to the development of more efficient foliar Zn fertilizers.

## Teaser

Synchrotron-based X-ray analysis of living wheat leaves showed that foliar-applied ZnEDTA is taken up as a ligand complex, and typical Zn agricultural application rates may induce localized toxicity

## Introduction

Nearly half of the world’s agricultural soils contain inadequate levels of available zinc (Zn), resulting in Zn deficiency in one-third of the global human population (Alloway, 2008*a*; International Zinc Association, 2010). Zn deficiency in humans is particularly problematic for women and children in developing countries (Kumssa *et al.*, 2015) where the majority of food intake consists of cereal grains (Prasad, 1996; Zou *et al.*, 2012). In staple food crops such as cereals, low concentrations of Zn are caused not only by low soil concentrations but also by the low bioavailability of Zn in soil (Alloway, 2008*a*).

It was recently proposed that the agricultural sector provides the only ‘sustainable solution to Zn deficiency in humans globally’ (Cakmak *et al.*, 2017). Soils with low concentrations of available Zn can be improved by adding Zn fertilizers, but this strategy can be ineffective and expensive in highly Zn-fixing soils (Graham and Rengel, 1993). Therefore, to avoid these issues, but still increase grain Zn concentrations, foliar Zn application can also be used (Cakmak, 2008; Zou *et al.*, 2012; Prasad *et al.*, 2014). This method can supplement the nutrient supply, overcoming the problem of low availability of soil nutrients. However, the factors that influence the effectiveness of foliar application are not well understood (Fernández and Eichert, 2009), including how the chemical form in which the nutrient is applied affects its uptake, translocation, and overall efficacy.

Zinc sulphate (ZnSO_4_) is the most commonly used inorganic form of soil-applied Zn fertilizer, whereas ZnEDTA is the most commonly used chelated source (Alloway, 2008*b*). In some soils, the availability of chelated Zn is at least double that of ZnSO_4_; however, it is also 5–10 times more expensive (Boawn, 1973; Karak *et al.*, 2005; Alloway, 2008*b*). When ZnSO_4_ and ZnEDTA fertilizers are used for foliar application, it is unclear which treatment is more efficacious; results vary depending on what parameter is used to define ‘agricultural efficacy’. Some studies have shown that foliar-applied ZnEDTA is more effective than ZnSO_4_ for increasing grain yield if applied before the tillering stage (Brennan, 1991). However, when dry shoot biomass was used as the measure of agricultural effectiveness, Haslett *et al.* (2001) found a minimal difference between foliar-applied ZnEDTA and ZnSO_4_. When comparing the effects of ZnEDTA and ZnSO_4_ on Zn grain concentration in different plant species, the results are also inconclusive. For example, in rice (*Oryza sativa* L.), the increase in the grain Zn concentration was significantly higher for ZnSO_4_ than for ZnEDTA (*P*<0.05) (Wei *et al.*, 2012), whereas in chickpea (*Cicer arietinum* L.), ZnEDTA resulted in a higher Zn seed content (Kayan *et al.*, 2015).

These past studies are difficult to compare directly as each study used different plants species (at different growth stages) under various environmental conditions (Fernández and Brown, 2013). Furthermore, the experimental methods that were used—total Zn content analysis using inductively coupled plasma mass spectrometry (Wei *et al.*, 2012) or atomic absorption spectroscopy (Brennan, 1991), and grain or crop yield (Brennan, 1991)—are not ideally suited to understanding the details of foliar absorption, translocation, and speciation of the different sources of Zn. As a result, the pathways and mechanisms of micronutrient uptake through leaves remain poorly understood, and there is uncertainty regarding the translocation and speciation of nutrients once they penetrate the leaf surface (Fernández, 2013). This lack of knowledge limits the refinement of fertilization strategies and the development of more effective formulations.

This study aims to provide detailed information on the mobility, distribution, and speciation of foliar-applied Zn in wheat using ^65^Zn-spiked solutions and state-of-the-art synchrotron techniques. Specifically, the objectives of this study are to: (i) assess and quantify the mobility of Zn applied to leaves as ZnSO_4_ or ZnEDTA using a radioisotope tracing technique and synchrotron-based X-ray fluorescence microscopy (XFM); and (ii) identify the chemical speciation of Zn, within leaf tissues to which Zn has been applied in ionic or chelated form (ZnSO_4_ and ZnEDTA, respectively) using X-ray absorption spectroscopy (XAS).

Ultimately, this experiment will test the hypotheses that ZnEDTA will be taken up more readily than ZnSO_4_ and be more mobile, and that ZnEDTA will be taken up and redistributed as the chelated complex.

## Materials and methods

### Plant growth in nutrient solutions

For the XAS experiments, wheat (*Triticum aestivum* cv Shield) plants were grown hydroponically in a naturally lit glasshouse with a median temperature of 21 °C. For the radioisotope experiment, plants were grown in a growing chamber (Conviron A1000) with a 16/8 h day/night cycle at 20 °C/16 °C, respectively, and humidity of 60%. Shield was used as it is a commercially important cultivar in Australia and is a double haploid wheat variety, making it relatively homogenous. Seeds were germinated for 4 d in 50 ml open-ended plastic cylinders filled with rockwool (a growing substrate to support the seedlings), then covered with black tape, and the cylinders were inserted in pots filled with 760 ml of deionized water. Following germination, the deionized water was replaced with a nutrient solution comprising: 1.0 mM KNO_3_, 1.0 mM Ca(NO_3_)_2_, 0.457 mM MgSO_4_, 0.1 mM KH_2_PO_4_, 1.0 μM MnCl_2_, 3 μM H_3_BO_3_, 1 μM (NH_4_)_6_Mo_7_O_24_, 1 μM, ZnSO_4_, 0.2 μM CuSO_4_, and 60 μM Fe(III)-EDTA (Li *et al.*, 2008), 0.0336 mM Na_2_SiO_3_, stabilized at pH 6 with 2 mM MES buffer, and aerated continuously.

### Uptake and translocation of zinc using radioisotopes

Two foliar fertilizers, ZnSO_4_ and ZnEDTA, were applied as aqueous solutions at the five-leaf growth stage (GS15) (Zadoks *et al.*, 1974). Each treatment was applied to a separate leaf and there were four replicates per treatment (i.e. four leaves per treatment). Both treatments had a Zn concentration of 1000 mg l^–1^, typical of agricultural Zn foliar sprays (Cakmak and Kutman, 2018), and were labelled with ^65^Zn at a rate of 2 kBq l^–1^. To ensure that the ZnEDTA was properly labelled with the Zn radioisotope, ^65^Zn solution was added to the ZnSO_4_ solution before EDTA was added. Both fertilizer solutions were adjusted to pH 6 using 100 mM NaOH and contained Tween-20 (0.05%, v/v) as a surfactant. Prior to fertilizer treatment, the leaves were marked with a permanent marker 20 mm from the tip. Three days later, the plants were removed from the growing chamber and the leaves were dipped in the fertilizer solutions up to this mark for 30 s. Plants were then immediately returned to the growing chamber. After 24 h, the fertilizer solutions had dried out on the leaf, and the leaf tip was cut from the leaf where it had been marked. The remainder of the leaf was then cut into fragments to detect the movement of applied Zn away from the application area. The first fragment was cut 3 mm from the point of application (i.e. 23 mm from the tip), followed by another nine fragments collected as follows: three fragments at 3 mm, two fragments at 4 mm, and four fragments at 5 mm (10 sections in total).

The ^65^Zn activity of the samples was analysed using gamma spectroscopy (Perkin Elmer 2480 WIZARD^2®^ Automatic Gamma Counter). Each leaf fragment was placed in a vial which was then placed in a gamma counter. The total amount of Zn taken up and translocated was calculated using the specific activity of Zn in the radiolabelled ZnEDTA and ZnSO_4_ solutions. The detection limit (*L*_D_), in counts, was determined using Currie’s formula (Currie, 1968):

$${\text{L}}_{\text{D}}=\text{3}.{\text{29}}_{\sigma \text{B}}$$

where σ_B_ is the standard deviation of the blank count rate, and the probabilities of Type I and Type II errors are assumed to be equal (α=β=0.05).

### Distribution and translocation of zinc using X-ray fluorescence microscopy (XFM)

**Fig. 2.**
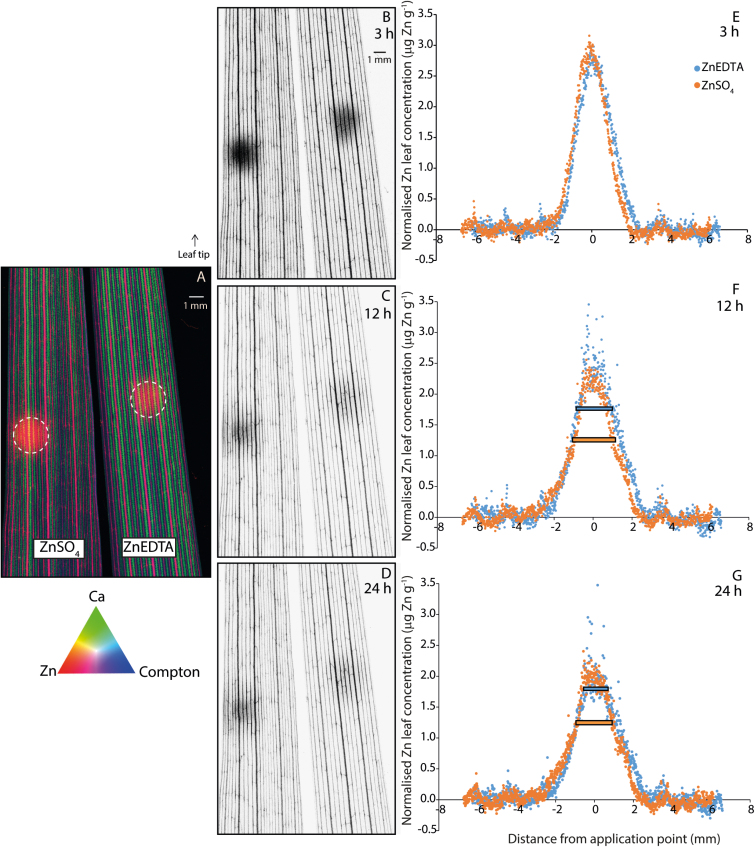
XFM time series experiment (XFM Experiment 1) on living wheat leaves (A–G). The Zn was applied to the leaves at *t*=0 h for a total of 3 h, with the unabsorbed Zn remaining in the droplet removed before analysis, where the dashed circle on the leaves (A) shows the size of the droplet. The XFM scans were conducted after 3 h (i.e. immediately after removal of the remaining unabsorbed Zn) (A, B), 12 h (9 h after removal) (C), and 24 h (21 h after removal) (D). In (A), the data are presented as a tricolour image, showing the elemental distribution of Zn (red), Ca (green), and Compton (blue) 3 h after droplet application of ZnSO_4_ and ZnEDTA (A). The data in (B), (C), and (D) are greyscale images. Data shown in (E), (F), and (G) correspond to the data extracted from (B), (C), and (D). After 12 h and 24 h, the distribution of ZnSO_4_ was broader than that of ZnEDTA, as indicated by the horizontal bars inserted at 50% of the maximum Zn concentration (F and G). XFM data were normalized to the background concentrations of Zn in leaves. For each image, the leaf exposed to ZnSO_4_ is on the left and the leaf exposed to ZnEDTA is on the right, with the size of the imaged area being 13.5 mm×20 mm.

The aim of the first XFM experiment (XFM Experiment 1) was to investigate the uptake and translocation of foliar-applied Zn. Specifically, we applied Zn to the leaves for 3 h before removing the unabsorbed Zn remaining in the droplet, with subsequent changes in the tissue distribution of Zn then examined over time. In this time-resolved experiment, the same fertilizers were used as for the radioisotope experiment (ZnSO_4_ and ZnEDTA, 1000 mg Zn l^–1^). One treatment was applied per leaf. One 5 µl droplet of each fertilizer (~2.5 mm in diameter, Fig. 2A) was applied on the central vein of the adaxial side of the youngest fully expanded leaf (YFEL) while still attached to the plant. The point of application was then marked with a permanent marker.

To minimize evaporation of the droplet, leaves (still attached to the plant) were placed in covered Petri dishes containing moistened filter paper for 3 h. During this 3 h exposure period, the droplets remained as a liquid and did not dry out. After 3 h, and immediately prior to the first XFM scan, unabsorbed fertilizer was removed from the leaf surface by gently wiping with KimTech wipes and sequentially rinsing with 2% nitric acid (HNO_3_), 3% ethanol, and deionized water (Du *et al.*, 2015). The XFM scans of the sample leaves were collected by mounting the plants, still growing in the tube holding the nutrient solution, on the back of a sample holder and gently bending the appropriate part of the leaves in an area that could be positioned, unimpeded, in the path of the X-ray beam. To mount the samples on the sample holder, leaves were partially covered in Ultralene^®^ film (4 µm thick). Scans were carried out after 3, 12, and 24 h (i.e. immediately upon removal of unabsorbed Zn, 9 h after droplet removal, and 21 h after droplet removal, respectively). For the first two scans (*t*=3 h and *t*=12 h), the analysed leaves were still attached to the plant, whereas, immediately prior to the last scan, the sample leaves were detached.

The sample leaves were scanned at the XFM beamline at the Australian Synchrotron in Melbourne, Australia as described previously (Du *et al.*, 2015). Briefly, single energy X-rays were selected using a Si(111) monochromator, and two Kirkpatrick–Baez mirrors were used to form a 2 × 2 μm^2^ focus on the sample (Paterson *et al.*, 2011). X-ray fluorescence emitted by the sample (and subsequent elemental maps) was collected using an incident energy of 12 900 eV and a 384-element Maia detector, in backscatter geometry. The sample leaf was analysed continuously horizontally with a sampling interval of 12 μm and a vertical step size of 12 μm. To avoid damaging the sample during XFM scanning, the transit time for each pixel was 3 ms (Lombi *et al.*, 2011). The X-ray fluorescence (XRF) spectra were analysed using GeoPIXE (Ryan and Jamieson, 1993; Ryan, 2000) and quantification was carried out as described previously (Lombi *et al.*, 2011).

In the second XFM experiment (XFM Experiment 2), the veinal and interveinal distribution of Zn, applied as ZnSO_4_, was investigated. Zn fertilizer was applied to the leaves using the same protocol as used in the first XFM experiment. Leaves were left for 6 h in Petri dishes in the presence of moistened filter paper, with the droplets again remaining as a liquid during the exposure period. The droplets were removed after 6 h, again using 2% nitric acid (HNO_3_), 3% ethanol, and deionized water. The leaves were excised and mounted on a sample holder using Ultralene film. The hydrated leaves were then scanned at room temperature at Sector 13-IDE of the Advanced Photon Source (APS) in Chicago, USA. The electron storage ring operated at 7 GeV in top-up mode, with the X-ray source being a 72 pole, 35 mm period undulator. A cooled Si(111) monochromator and Kirkpatrick–Baez focusing mirrors were used to obtain a monochromatic beam focused onto the specimen. Elemental maps were collected using an incident energy of 10 500 eV and a 4-element Vortex detector. The sample leaf was mounted at a 45° angle relative to the incident beam and analysed continuously horizontally with a sampling interval of 8 μm and a vertical step size of 8 μm. The transit time for each pixel was 10 ms. The spectra were analysed using GSE MapViewer. Leaves treated with ZnEDTA were not investigated in this experiment. However, similar data (although at a lower resolution) were extracted from an XFM scan of a ZnEDTA-treated leaf in XFM Experiment 1.

### Speciation of absorbed zinc using X-ray absorption spectroscopy (XAS)

XANES analysis was used to determine the speciation of Zn in leaves following foliar application of ZnEDTA and ZnSO_4_. The XAS analysis was conducted at the XAS Beamline at the Australian Synchrotron. The X-ray beam was tuned with an Si(111) monochromator in the energy ranges of 9459–9639 eV (3 eV steps) for the pre-edge and 9639–9709 (in 0.25 eV steps) for the edge region (the post-edge extended to a k of 10, scanned at 0.05 k steps). The beam size was adjusted to ~1.2 × 0.7 (H×V) mm. Sample spectra were collected in fluorescence mode with a 36-element solid-state Ge detector. At the same time, the XANES spectrum of a metallic Zn reference foil was collected in transmission mode and this spectrum was used to energy calibrate the sample spectra. The spectra were energy normalized and background corrected using the Athena software package (Ravel and Newville, 2005). Linear combination fitting (LCF) of the sample spectra was performed using the Athena software in the fitting range –30 eV to +100 eV relative to the Zn K_α_-edge.

Fertilizer droplets were applied to the leaves, sealed in a moistened Petri dish as described in the XFM experiments, and left for 24 h. Again, it was noted that during this 24 h experimental period, the droplets remained as a liquid and did not dry out. Leaves were then washed using the same procedure as for the XFM experiments. The leaf sample was mounted vertically on a Perspex holder with a 13 mm diameter window using two pieces of Kapton tape. When placing the leaf in the sample holder, a small strip of aluminium foil was placed next to the leaf where the droplet had been applied, to pinpoint the fertilizer application area during the scans. The sample was then plunged into liquid nitrogen and rapidly mounted on a cryostat maintained at 10 K using liquid He. The leaf was scanned along the longitudinal axis, using an incident energy above the Zn K_α_-edge (10 040 eV) to obtain a line scan of the Zn distribution. To determine the Zn speciation at different distances from the point of application, XANES spectra were collected at various positions based on the signal intensity. First, the background and maximum Zn signal intensities were determined. The background signal was located between 3 mm and 5 mm from the centre of the line scan, whereas the maximum signal intensity was in the centre of the line scan. XANES maps were collected at these positions, as well as at positions at 10, 25, and 50% of the intensity between these two positions. The total length of the line scans was between 8 mm and 10 mm. Due to the high variation in background Zn concentrations between leaves, a separate untreated leaf is not suitable for quantifying background Zn in treated leaves. However, XFM analysis of an untreated leaf was performed to characterize the distribution of background Zn (see Supplementary Fig. S1 at *JXB* online).

Using the same methods as described above, seven Zn reference materials were also measured at the XAS beamline: Zn-citrate, Zn-phytate, Zn-cysteine, Zn-polygalacturonate, Zn-histidine, ZnSO_4_, and ZnEDTA. All prepared reference materials had a pH of 6.5 (adjusted using 0.1 M NaOH), and a nominal concentration of 350 mg Zn l^–1^, with the exceptions of Zn-polygalacturonate (180 mg Zn l^–1^), and ZnSO_4_ and ZnEDTA (500 mg Zn l^–1^). All of these compounds (excluding ZnSO_4_) were used for the LCF of all sample spectra. See Supplementary Protocol S1 for the preparation methods used for all Zn standards.

### Statistical analysis

Welch’s two-sample *t*-test was undertaken using R version 3.4.0 to determine the mobility of different Zn treatments in wheat leaves. This parametric test assumes that the two populations (ZnEDTA and ZnSO_4_) have normal distributions. Significant differences were determined at a level of *P*<0.05.

## Results

### Mobility and translocation of zinc using radioisotopes

**Fig. 1.**
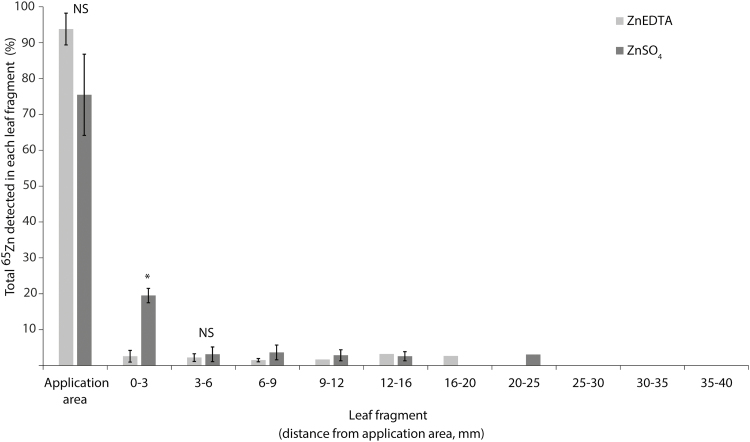
Distribution of ^65^Zn in leaves following foliar application of ZnEDTA and ZnSO_4_. The calculated mass of ^65^Zn in each leaf fragment is presented as the mean percentage (±1SD, *n*=4) of the total mass of ^65^Zn in the leaf (i.e. the total of all columns equals 100%). The asterisk indicates a significant difference between ZnEDTA and ZnSO_4_ for that leaf fragment (*P*=0.0009); NS, not significantly different. Statistical tests could only be applied to the first three leaf fragments (i.e. application area of 6 mm) due to unequal sample sizes for the remaining fragments as ^65^Zn was below the detection limit in some samples. Therefore, statistical tests would not be conclusively, or reliably, detected. The first bar represents the 20 mm of leaf tip that was dipped in fertilizer. Error bars are not shown for leaf fragments where ^65^Zn was only detected in one or two of the four replicates.

Zinc applied to leaves as radiolabelled ^65^ZnSO_4_ was slightly more mobile compared with ZnEDTA (Fig. 1). In the leaf section adjacent to the application point (0–3 mm), there was a significantly greater proportion (*P*=0.0009) of total ^65^Zn in ZnSO_4_-treated leaves compared with ZnEDTA-treated leaves (20% of the total ^65^Zn for ZnSO_4_ compared with 3% for ZnEDTA) (Fig. 1). In agreement with this observation, more ^65^Zn appeared to remain at the application point in ZnEDTA-treated leaves (93.8 ± 4.4%) compared with the ZnSO_4_-treated leaves (75.5 ± 11.3%) (Fig. 1, *P*=0.06). For the leaf fragment 3–6 mm from the application point, there was no significant difference between fertilizer treatments (*P*=0.61) (Fig. 1). ^65^Zn was not detected at distances ≥20–25 mm from where ZnEDTA and ZnSO_4_ were applied, or in any of the control leaves. Note, as the ^65^Zn present on the surface of the leaves was not removed, Zn absorption was not directly investigated in this experiment.

### Spatial distribution and translocation of zinc using XFM

Based on XFM analysis, the background concentrations of Zn in the YFELs of ZnEDTA-and ZnSO_4_-treated plants were 14 mg Zn kg^–1^ and 18 mg Zn kg^–1^, respectively. These concentrations are based on the average Zn concentration in areas as far from the applied droplet as possible (determined by the size of the scanned area). The concentrations in these areas did not change from 3 h to 24 h, so it is most likely that it is background Zn, and not Zn moving away from the applied droplet. XFM Experiment 1 investigated changes in the distribution of Zn in leaf tissues to which Zn had been applied for 3 h before being removed. Areas of high Zn concentration—where Zn treatments were applied to leaves— were clearly visible as circular ‘hot spots’ in the XFM images (Fig. 2A–D). The XFM data showed that the applied Zn did not cause any physical damage to the leaf (i.e. leaf scorch). Leaf scorch, and the consequent loss of water, reduces the thickness of the leaf, and therefore thinner areas of the leaf would appear as a darker colour in the Compton scattering map. As this was not observed in the leaf tissue under the fertilizer application area, it is unlikely that Zn damaged the leaf.

The first scan was collected 3 h after applying the droplet (and immediately after removing the unabsorbed Zn from the leaf surface). In this first scan, the ZnSO_4_ spot appeared slightly more intense than the ZnEDTA spot (Fig. 2B), suggesting a higher absorption of Zn when applied as ZnSO_4_. However, in the scans collected 12 h and 24 h after fertilizer application (i.e. 9 h and 21 h after the unabsorbed Zn had been removed from the leaf surface) (Fig. 2C and D, respectively), the ‘hot spots’ for both treatments were of a similar intensity. Veins of the leaves were also visible as parallel lines in XFM images. To support our hypothesis that the measured Zn was internalized and not on the leaf surface, XFM scans of freeze-dried transverse leaf cross-sections were collected (Supplementary Fig. S2).

While the XFM images were useful for understanding the general distribution of Zn in leaves, visual appearance alone could not give conclusive results regarding the differences in Zn uptake between treatments. Therefore, data were extracted from the XFM images and further analysed (Figs 2E–G). After normalizing these data to compensate for differences in background Zn concentrations, Zn absorption after 3 h was found to be similar between treatments. However, after 12 h and 24 h (i.e. 9 h and 21 h after the unabsorbed Zn had been removed from the leaf surface), Zn applied as ZnSO_4_ was slightly more mobile than Zn applied as ZnEDTA based on two observations: (i) more Zn remained at the application point in the ZnEDTA-treated leaf; and (ii) after 12 h and 24 h, the distribution of Zn was broader in the ZnSO_4_-treated leaf compared with the ZnEDTA-treated leaf (Fig. 2F, G). Therefore, despite the visual similarities between XFM images for both treatments, detailed analysis indicates that Zn applied as ZnEDTA was slightly less mobile than Zn applied as ZnSO_4_. In terms of the rate of Zn movement, Zn from the ZnSO_4_ treatment was more mobile between 3 h and 12 h than between 12 h and 24 h after application (Supplementary Figs S3, S4A). However, for the ZnEDTA treatment, Zn mobility appeared relatively consistent between 3, 12, and 24 h (Supplementary Figs S3, S4B).

**Fig. 3.**
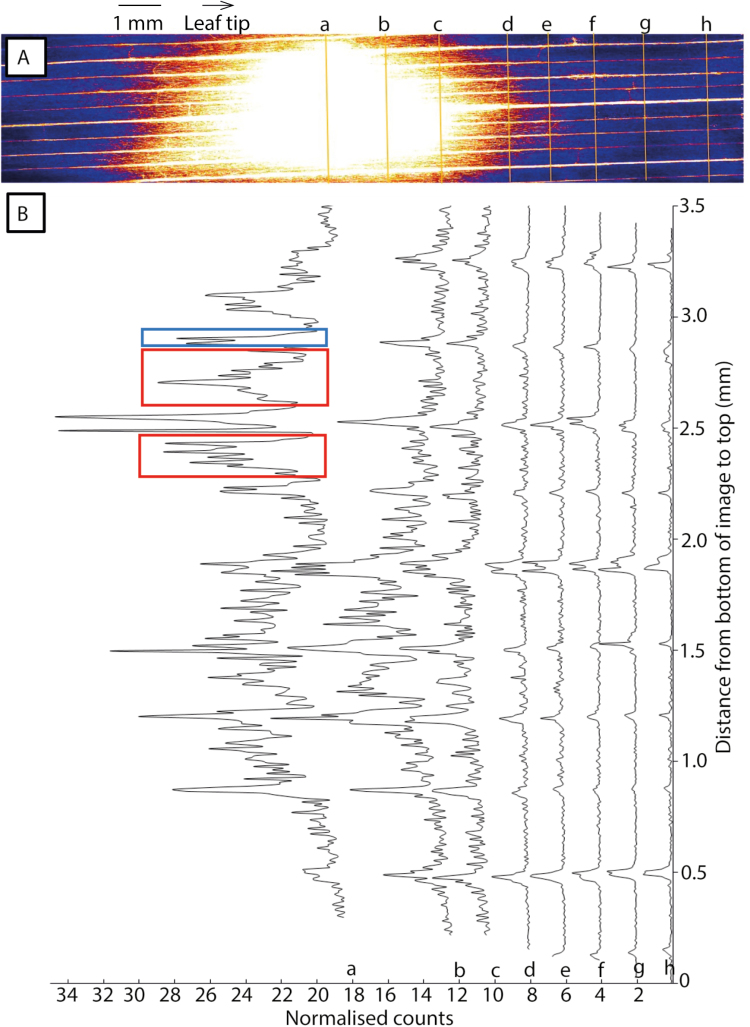
XFM image (A) of a leaf treated with a droplet of ZnSO_4_ and removed after 6 h (XFM Experiment 2). Line scans (B) were collected across the leaf at locations shown by the yellow lines in the XFM image (transects a–h). The red dashed rectangles indicate intervein areas with a high Zn concentration. The intensity of these peaks significantly decreases moving away from the hot spots (i.e. transect b), whereas similar Zn concentrations in the veins (blue square) do not decrease to the same extent in transect b.

In XFM Experiment 2 (performed *in situ* using hydrated, excised leaves), transverse line scans were collected across the leaf (i.e. perpendicular to the central vein; Fig. 3). Peaks in the XFM transect showed that Zn accumulated in veins (transects d–h Fig. 3). Zinc was also observed in the smaller transverse veins interconnecting the main longitudinal cross-veins. Moving away from the application point, the amount of Zn decreased more quickly from the interveinal area than from the veins (circled areas in Fig. 3B), suggesting that Zn was translocated through the veins. The same Zn distribution pattern was observed in a ZnEDTA-treated leaf (Supplementary Fig. S5) collected from Experiment 1, where a high number of counts was detected in the veins of the leaf tissue underneath the droplet application site (Supplementary Fig. S5 transects a–c), and then a decrease moving away from the application site (Supplementary Fig. S5 transects d–h). The split peaks in some transects suggest that Zn was translocated within multiple vessels within a single vein, for example the split peaks located 1.9 mm from the bottom of the image for transects d–h (Fig. 3B). The variations in sample thickness were accounted for when calculating Zn concentrations.

### Speciation of absorbed zinc using XAS

First, we compared the XANES spectra of the seven standard compounds, which were found to show some marked differences (Supplementary Fig. S6). These differences were determined by comparing (i) the energy of the white-line peak; (ii) the height of the white-line peak; and (iii) spectral features of each standard. The white-line peak corresponded to an energy of 9665 eV for Zn-phosphate, 9667 eV for Zn-phytate, Zn-histidine, Zn-citrate, Zn-EDTA, and Zn-polygalacturonate, and 9668 eV for Zn-cysteine. This shift to higher edge energies can be explained by the higher electronegativity of S (the thiol group in Zn-cysteine) compared with P (Zn-phosphate). Compared with the white-line energies, the heights of the peaks showed more variation between standards. The white-line peak height decreased in the order Zn-citrate >ZnEDTA >Zn-polygalacturonate >Zn-phytate ~ Zn-phosphate >Zn-cysteine ~ Zn-histidine. Zinc-phytate and Zn-phosphate were distinguishable from each other not only by their differences in peak energy, but also by spectral differences, such as the broader peak for Zn-phytate (Supplementary Fig. S6). Similarly, for Zn-cysteine and Zn-histidine, the less well defined post-edge features of the Zn-histidine spectrum made it distinguishable from Zn-cysteine.

**Fig. 4.**
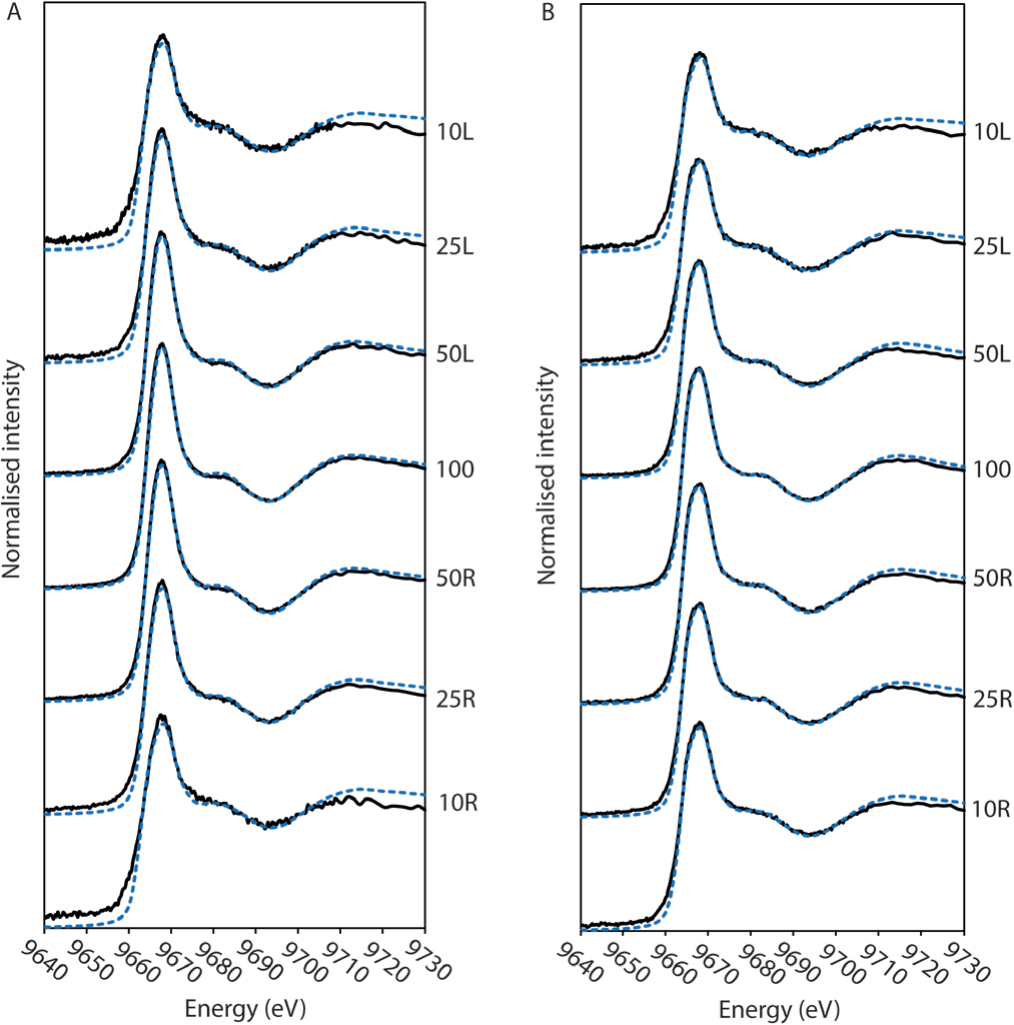
Zinc K_α_-edge XANES spectra of ZnSO_4_ (A) and ZnEDTA- (B) treated leaves. The secondary *y*-axis indicates the position on the leaf where the spectra were collected with respect to maximum signal intensity (100). XANES maps were collected at 50, 25, and 10% of the maximum signal intensity along the longitudinal axis of the leaf from left (L) to right (R), towards the leaf tip and stem, respectively. The experimental fit (black solid line) and linear combination fit (LCF) (blue dashed line) of reference spectra are shown for each leaf sample. Normalized Zn K_α_-edge XANES spectra for the standard compounds used in the LCF are shown in Supplementary Fig. S6.

Next, we compared the XANES spectra for the plant tissues. It was noted that not only were the Zn K_α_-edge XANES spectra collected from ZnSO_4_- and ZnEDTA-treated leaves different from each other (Supplementary Fig. S7), but these spectra also differed from the spectra collected at a location on the leaf where the maximum signal intensity was 10% of that at the site of application (Fig. 4; Supplementary Fig. S8); that is, approaching background concentrations. Indeed, these spectra had a lower peak intensity (Supplementary Fig. S8) and less defined structural features, and the ZnSO_4_ spectrum had a higher peak intensity than that of ZnEDTA (Supplementary Fig. S7).

**Fig. 5.**
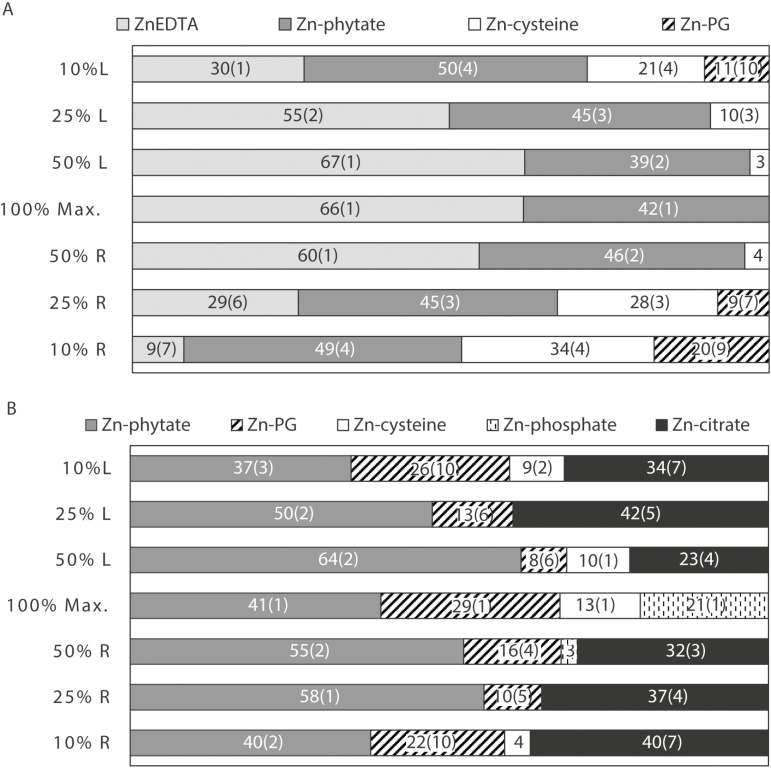
Distribution of Zn species in wheat leaves treated with foliar applications of ZnEDTA (A) or ZnSO_4_ (B). The maximum signal intensity (100% maximum) corresponds to the fertilizer application point. XANES maps were collected at 50, 25, and 10% of the maximum signal intensity along the longitudinal axis of the leaf from left (L) to right (R) (e.g. from 10% L to 10% R of the fertilizer application point, respectively). The percentage variation in the calculated values (standard errors) is shown in parentheses. For percentage contributions that were <5%, the associated standard errors were <1% and are shown in Supplementary Table S1 for clarity.

Using LCF, it was predicted for the background tissue that the majority of the Zn was bound to cysteine (73–86%), with the speciation of Zn in the background tissue being the same regardless of the form in which Zn was applied to leaves (Supplementary Fig. S9). Zinc-phytate was predicted to be the second most abundant Zn species (12–32%) in the background leaf tissues (Supplementary Fig. S9). Using predictions from LCF, some similarities in speciation were found between treatments and the background; for example, some Zn was associated with phytate in all samples leaves. Compared with other Zn species, the proportion of Zn-phytate was relatively consistent at different locations along the leaves and ranged from 37% to 64% for ZnSO_4_ and from 39% to 50% for ZnEDTA (Fig. 5).

For both treatments, Zn speciation at the point of maximum signal intensity (i.e. the point of fertilizer application) was different from that at other points on the leaf. For example, in ZnSO_4_-treated leaves, 21% of Zn was predicted to be associated with phosphate at the point of application but not at any other scanned positions on the leaf (with the exception of a minor amount in one location) (Fig. 5B). Conversely, Zn-citrate was predicted to be a significant component (≥23%) at all locations analysed, except at the point of application.

For ZnEDTA-treated leaves, Zn-phytate and ZnEDTA were the only two species predicted to be present in the leaf tissues at the point of application (Fig. 5A). Again, the proportion of Zn-phytate was relatively constant between all scanned locations, whereas the proportion of ZnEDTA decreased from 66% to 9% moving away from the application point. There was also a concomitant increase in the proportion of Zn bound to carboxyl (polygalacturonate) and thiol (cysteine) groups.

## Discussion

### Zinc has limited mobility in wheat leaves regardless of the form in which it is applied

Results from the radiolabelled experiment and XFM analyses showed that foliar-applied Zn was relatively immobile in leaves. Radiolabelled Zn was not detected beyond 20 mm from where ^65^ZnEDTA was applied and 25 mm from where ^65^ZnSO_4_ was applied (Fig. 1). Zinc appeared even less mobile in our XFM analyses where tissue Zn concentrations decreased rapidly at >2.5 mm from the edge of the fertilizer droplet for both treatments. This slight difference in mobility may be due to the sensitivities of both the techniques and instruments used for the analyses, and the large Zn background present which makes the XFM method intrinsically less precise. On the other hand, the XFM method used here allowed us to map precisely the diffusion of Zn away from the point of application on the same leaves over time. To our knowledge, this is the first application of time-resolved elemental imaging in plants using this synchrotron technique. The limited mobility of foliar-applied Zn has been attributed to its poor leaf penetration, and high binding capacity of Zn to leaf tissues (Marschner, 1995; Montalvo *et al.*, 2016); the conditional mobility of Zn in the phloem is likely to be less important (Fernández and Brown, 2013)

Although comparable studies using wheat plants could not be found, our results are in general agreement with previous studies using other plant species that have shown limited mobility of foliar-applied Zn. For example, when Zn(NO_3_)_2_ was applied as a droplet (400 mg l^–1^) to the YFEL of tomato (*Solanum lycopersicum*) plants, Zn concentrations in interveinal tissue decreased to background levels within 1.5–3 mm of the edge of the droplet (Du *et al.*, 2015). Zhang and Brown (1999) investigated the distribution of foliar-applied Zn in pistachio (*Pistachio vera* L.) using a stable Zn tracer (^68^Zn in the form of ZnO). At 25 d after sectioning ^68^Zn-treated leaves into three parts (apical, middle, and basal), 84% of applied ^68^Zn remained in the midsection (where Zn was applied) and only 4% was detected in the apical sections and 13% in the basal sections. Using synchrotron-based XFM, the mobility of foliar-absorbed Zn in the veins of sunflower (*Helianthus annuus*) and tomato was limited to 0.96 ± 0.26 mm and 0.88 ± 0.13 mm (mean, *n*=4), respectively (Li *et al.*, 2017). Zinc was less mobile in the interveinal tissues for both species (Li *et al.*, 2017).

To the best of our knowledge, the current study is the first to compare and quantify the mobility of foliar-applied ZnEDTA and ZnSO_4_ in living wheat leaves at such fine spatial resolution (micromolar to millimolar range). Using similar XFM methods to the current study, Tian *et al.* (2015) showed that foliar-applied Zn (200 mg Zn l^–1^) was more mobile in the phloem of sunflower plants when applied with EDTA compared with ZnSO_4_ alone. In dwarf pea plants (*Pisum sativum* cv Douce Provence), translocation of Zn away from the treated leaf to other plant parts was similar for both fertilizers, where 4.9% of the Zn from ZnEDTA and 4.7% of the Zn from ZnSO_4_ was translocated after 24 h (Ferrandon and Chamel, 1988). In the current study, although Zn was relatively immobile in leaf tissue, both experiments showed that ZnSO_4_ was slightly more mobile than ZnEDTA, contradicting our original hypothesis that ZnEDTA would have greater mobility.

Following leaf surface penetration, inorganic nutrients may be transported through apoplastic or symplastic spaces (Yumei *et al.*, 2014). Based on previous studies (Fernández and Brown, 2013), we hypothesized that the abundance of negatively charged sites in the apoplast (e.g. cell walls) would limit the translocation of positively charged Zn^2+^, whereas chelated Zn would be ‘protected’ from sorption. The results did not support this hypothesis, indicating that the adsorption of Zn^2+^ at negatively charged sites is not the primary factor controlling Zn mobility in leaves. This is analogous to the uptake and mobility of Zn^2+^ in plant roots where the ion is taken up efficiently and does not strongly bind to the cell wall or membrane components (Hart *et al.*, 1998). Therefore, additional factors are likely to have influenced Zn mobility such as (i) the dominance of other transport pathways (i.e. symplastic transport); (ii) a proportion of Zn may have been rapidly released from the ZnEDTA complex and thus its mobility would follow that of Zn^2+^; or (iii) Zn^2+^ may have been complexed and stabilized by other compounds after penetrating the cuticle. Further investigation is required to determine what effect, if any, (i) had on Zn mobility. In the current study, the influence of (ii) and (iii) was investigated using Zn XAS chemical speciation analysis.

### Foliar-applied zinc is distributed rapidly in wheat leaves and moves towards the veins

Although limited, the translocation of Zn occurred quickly for both treatments (<24 h). This is in agreement with previous studies which have reported that a substantial amount of foliar-applied ^65^Zn was transported to other plant parts within 48 h (Haslett *et al.*, 2001). In our time-resolved XFM experiment (XFM Experiment 1), there appeared to be more movement of Zn between 3 h and 12 h than between 12 h and 24 h for both Zn treatments (Fig. 2B–G). It is unknown why this occurred, but both the nutritional status of the plant and the complexation of Zn may have played a role. In regard to nutritional status, the background concentrations of Zn in the YFELs of ZnEDTA- and ZnSO_4_-treated plants were 14 mg Zn kg^–1^ and 18 mg Zn kg^–1^, respectively. This indicates a slight Zn deficiency where concentrations >20–24 mg Zn kg^–1^ are considered adequate for this growth stage (Wilhelm and Davey, 2016). However, further investigation is required to determine if this had a significant effect on Zn translocation, as conflicting results have been reported as to whether Zn deficiency promotes or limits Zn translocation (Longnecker and Robson, 1993; Erenoglu *et al.*, 2002; Du *et al.*, 2015).

Analysis of the XANES data, which is further discussed in the following paragraphs, suggests that the second factor (formation of Zn complexes) had an important effect on Zn mobility, and this may have been in response to Zn toxicity. Once Zn was taken up by the leaf, a small proportion of Zn may have been relatively mobile and moved quickly from the application site, whereas a larger proportion was bound by various ligands, greatly limiting Zn mobility. The speciation data, namely binding of Zn with phytate, citrate, and phosphate ligands, and the limited mobility of Zn suggest that the chemical speciation of Zn had considerable influence on the mobility of Zn, and, locally toxic concentrations of Zn may have occurred at the point of fertilizer application.

### The forms of Zn taken up differ between ZnEDTA and ZnSO_4_ foliar fertilizers, which also differ from the form of Zn in the background tissues of wheat leaves

The chemical speciation of Zn in leaf tissues was affected by both the form of Zn supplied and the distance from the fertilizer application point, and may explain the differences in Zn mobility observed for each treatment. At the fertilizer application point, ~40% of Zn in underlying tissues was present as Zn-phytate in both treatments (Fig. 5). This Zn species was the predominant form of Zn at the application point for leaves treated with ZnSO_4._ However, for ZnEDTA-treated leaves, ZnEDTA itself was the dominant Zn species (66%) at the application point (Fig. 5).

This is the first time, to the best of our knowledge, it has been shown that ZnEDTA penetrates the leaf surface and is redistributed in leaf tissue in chelated form. The uptake pathway of foliar-applied chelated metals is not well understood. However, it has been suggested that the leaf cuticle is the primary barrier for the absorption of foliar nutrients (Fernández *et al.*, 2017). While the uptake of hydrophobic compounds may simply occur through dissolution–diffusion processes, hydrophilic moieties (such as ZnEDTA and Zn^2+^) may cross the cuticle where it is damaged, or cross the leaf surface through epidermal structures such as the stomata, trichomes, specialized epidermal cells, or ‘aqueous pores’ (Rios *et al.*, 2016). While further investigation is required to understand the specific uptake pathway of hydrophilic chelated complexes, the results show that larger hydrophilic compounds such as ZnEDTA can cross the leaf surface.

At the site of fertilizer application, the speciation of Zn was different from that in the background tissues. Although Zn-phytate, ZnEDTA, and Zn-polygalacturonate were the dominant Zn species in leaves treated with Zn, for background tissues the predominant form was Zn-cysteine (>70%), with lesser amounts of Zn-phytate (≤32%) (Supplementary Fig. S9). In ZnSO_4_-treated leaves, only a minor proportion was predicted to be present as Zn-cysteine (<14%; Fig. 5) at the application point. Further, in ZnEDTA-treated leaves, Zn-cysteine was not detected at the application point. There are conflicting results regarding the fraction of leaf Zn present as Zn-cysteine. Terzano *et al.* (2008) found that ~50% of foliar Zn was associated with cysteine in rocket (*Eruca vesicaria* L. Cavalieri), whereas in cowpea (*Vigna ungui culata* L. Walp) grown in ZnCl_2_-spiked soil, Zn-cysteine was only identified in the nodules and seeds, not in the leaves (Wang *et al.*, 2013).

The observations above raise several important points regarding the form in which foliar applied Zn is taken up and its chemical speciation when transported in the leaf away from the application site. As stated above, there was a significant interaction between Zn and phytate in leaves exposed to both Zn treatments and for background Zn. Foliar application of ZnSO_4_ and ZnEDTA substantially increased the proportion of Zn associated with phytate (45% for ZnSO_4_ and 49% for ZnEDTA) compared with that in the control leaves (24%). Phytate is the primary storage form of P in cereals and legumes, comprising 40–85% of the total P in seeds and grains (Reddy *et al.*, 1982, 1989; Noack *et al.*, 2012). However, little is known about the contribution of phytate to total P in other plant parts under subtoxic conditions. Using ^31^P NMR spectroscopy, Noack *et al.* (2014) did not detect any phytate in the leaves of mature wheat plants; orthophosphate (85%) and glycerophosphate (15%) were the most abundant forms of P. In the seed, stems, and chaff, on the other hand, phytate was detected. The stability of the Zn^2+^-phytate complex is influenced by pH and the stoichiometry of the complex which in turn is affected by the initial Zn^2+^:phytate ratio (Crea *et al.*, 2008). In plants and soil, phytic acid can bind with metal ions to form stable insoluble complexes (Martin and Evans, 1986; Yan *et al.*, 2014). There is extensive literature on the bioavailability of P and Zn in soil and plant grains, yet it is uncertain how Zn toxicity affects P conditions, and, conversely, how high P concentrations affect Zn mobility in vegetative plant parts.

Zinc-polygalacturonate, Zn-citrate, and Zn-cysteine made a minor contribution to the overall speciation of Zn in Zn-treated leaves; however, their presence provides important insights into the fate of foliar-applied Zn. A substantial proportion of Zn was predicted to be associated with polygalacturonic acid in ZnSO_4_-treated leaves (8–29%), but only at three of the seven scanned locations in ZnEDTA-treated leaves (Fig. 5). Polygalacturonic acid is a major component of pectin (pectic polysaccharides), which is most abundant in plant primary cell walls and the middle lamella (Caffall and Mohnen, 2009). This suggests that when ZnSO_4_ is applied to wheat leaves, absorbed Zn^2+^ will bind to the negatively charged cell walls, whereas for ZnEDTA this pathway is less important. Homogalacturonan is one of the most abundant pectin biopolymers in cell walls and consists of linear chains of α-1,4-linked d-galacturonic acid (GalA) residues (Ridley *et al.*, 2001). In aqueous solution, it has been shown that Zn^2+^ can bind to GalA residues in pectin, and that the lower the degree of esterification in these residues the higher the Zn^2+^ binding capacity (Khotimchenko *et al.*, 2008).

Away from the point of ZnSO_4_ fertilizer application, Zn was predicted to be associated with citrate (23–42%) (Fig. 3). In this study, the citrate ligand can be considered representative of any carboxyl group of an organic acid (e.g. malate or oxalate). At neutral pH, the stability constant for Zn-citrate is higher than that for Zn- malate and Zn-oxalate, but, under acidic conditions, oxalate forms a stronger Zn complex (Jones, 1998). Therefore, the pH of cell vacuoles—where organic acids are primarily found (Wang *et al.*, 1992)—can influence the speciation of intracellular Zn in wheat leaves. The results are in agreement with previous studies which have shown a high proportion of leaf Zn to be associated with citrate in cowpea (Wang *et al.*, 2013) and in the hyperaccumulator Alpine Penny-cress (*Noccaea caerulescens*, formerly *Thlaspi caerulescens*) where large amounts of Zn can be stored with organic acids in the vacuoles of leaf epidermal cells (Küpper *et al.*, 1999; Frey *et al.*, 2000; Schneider *et al.*, 2013). Zn-citrate was not a significant component in the LCF of ZnEDTA-treated leaves. This may be attributed to the lower stability constants of Zn-citrate compared with ZnEDTA and/or that substantially less foliar-applied ZnEDTA is stored in and transported to cell vacuoles in wheat.

The stability of Zn complexes is likely to have had a strong influence on the speciation of Zn in leaf tissues. For example, in ZnEDTA-treated leaves, Zn-cysteine was not predicted to be present at the application site, but its predicted contribution increased to 34% away from this point. It is unclear why this occurred. One possibility is that Zn^2+^ is released from the EDTA complex when it is transported in the plant and preferentially binds to cysteine ligands. Alternatively, the proportion of ZnEDTA relative to other Zn chemical species may simply decrease further away from the application point. We believe the latter is the most likely scenario given the high stability constant of ZnEDTA (logK=17.5; Norvell, 1991). The high stability of this complex can affect the abundance of other Zn complexes, where those that have a lower stability constant—such as Zn-citrate (logK ~5; Wyrzykowski and Chmurzyński, 2010)—are less favourable. This may also explain why Zn-citrate was not detected in the ZnEDTA-treated leaf (Fig. 5). This chelation of Zn by organic ligands can act to decrease the transport of Zn in leaf tissue and restrict its translocation.

### High zinc concentrations in foliar sprays may determine zinc chemical speciation

The concentration of Zn in commercial foliar Zn sprays can be exceptionally high (up to 1500 mg l^–1^), potentially higher than the rate used in the present experiments (1000 mg Zn l^–1^). At these concentrations, Zn can cause leaf damage (scorch) under the area where the droplets sit. Therefore, localized toxicity may have occurred in the leaf given that Zn was absorbed relatively quickly and had limited mobility. This hypothesis is supported by the Zn chemical speciation data (Fig. 5).

Previous studies have shown that when various agronomic plant species are exposed to high Zn concentrations (40–300 μM) via the roots, a large proportion of Zn accumulates as Zn-phytate (van Steveninck *et al.*, 1994; Kopittke *et al.*, 2011). Both authors suggested that this may be a defence mechanism that acts to limit the transport of high concentrations of Zn to shoots. Therefore, the increased proportion of Zn-phytate observed in ZnEDTA-treated (39–50%) and ZnSO_4_-treated leaves (37–64%) compared with the control leaves (12– 32%) may be an important Zn detoxification mechanism where complexation with phytate decreases the mobility and bioavailability of Zn in leaves. Also supporting this hypothesis is the presence of Zn-phosphate (21%) at the ZnSO_4_ application point (where the highest concentration of Zn was observed). It has been suggested that this chemical species is important for the detoxification of high concentrations of Zn in plants. For example, in the leaves of rocket grown in Zn-contaminated soil, ~50% of Zn was predicted to be present as Zn-phosphate (Terzano *et al.*, 2008). Therefore, in the current study, plant toxicity defence mechanisms may have had a major influence, and possibly been the key determinant of, Zn chemical speciation in leaves.

### Conclusions

This study demonstrated the limited mobility of Zn in wheat leaves when applied as ZnEDTA and ZnSO_4_ foliar fertilizers. By using ^65^Zn radiolabelled foliar treatments, we were able to detect subtle differences in the mobility of ZnSO_4_ and ZnEDTA, where ZnSO_4_ was slightly more mobile in leaves. Synchrotron-based XAS analyses were used to determine the transformation and subsequent speciation of foliar-applied Zn. The ZnEDTA complex was taken up in chelated form and transported in this form most probably due to the high stability of this complex compared with organic acids. High concentrations of Zn in foliar fertilizers are likely to affect Zn chemical speciation and mobility strongly due to localized Zn toxicity in the plant leaf. Therefore, Zn foliar fertilizers with slower release rates may be advantageous as they could act to reduce toxicity on a local scale, thereby limiting the subsequent detoxification mechanisms that decrease Zn bioavailability. This study focused on the immediate uptake and behaviour of foliar-applied Zn; longer term plant studies are recommended to understand how Zn speciation affects the agronomic effectiveness of ZnEDTA and ZnSO_4_ foliar fertilizers.

## Supplementary data

Supplementary data are available at *JXB* online.

Protocol S1. Methods for the preparation of XAS standard compounds, XFM analysis of a transverse leaf cross-section, and XFM analysis of a ZnEDTA-treated leaf with corresponding transects.

Table S1. Distributions of Zn species in wheat leaves treated with foliar application of

ZnEDTA and ZnSO_4_, and the percentage variation in the calculated values shown in parentheses.

Fig. S1. X-ray fluorescence microscopy (XFM) image of an untreated leaf of wheat showing the presence of Zn in the veins of the leaf, but not in the interveinal areas.

Fig. S2. Transverse cross-section of a wheat leaf showing the internalization of Zn.

Fig. S3. X-ray fluorescence microscopy (XFM) images of wheat leaves showing the distribution of Zn following foliar application of ZnSO_4_ and ZnEDTA.

Fig. S4. Distribution of Zn in wheat leaves 3, 12, and 24 h after the foliar application of ZnSO_4_ and ZnEDTA from XFM data.

Fig S5. XFM image of a leaf treated with a droplet of ZnEDTA (1000 mg Zn l^–1^) and removed after 3 h.

Fig. S6. Zinc K_α_-edge XANES spectra of standard compounds used in the linear combination fitting of sample spectra.

Fig. S7. Zinc K_α_-edge XANES spectra showing the difference between the spectra for Zn in the leaf tissues at the site of fertilizer application following exposure to ZnSO_4_ or ZnEDTA.

Fig. S8. Zinc K_α_-edge XANES spectra showing the difference between speciation at the site of fertilizer application and at a location on the leaf where the maximum signal intensity is 10% of that at the site of application.

Fig. S9. The background distribution of Zn species in ZnSO_4_- and ZnEDTA-treated leaves as determined from LCF of the K_α_-edge XANES spectra.

Supplementary Material
