# Supplementary material for: Foliar application of zinc sulphate and zinc EDTA to wheat leaves: differences in mobility, distribution, and speciation
Source: J Exp Bot. 2018 Jun 21;69(18):4469–81. doi: 10.1093/jxb/ery236 (PMC6093386; doi:10.1093/jxb/ery236)
Supplement: Supplementary Material [file ery236_suppl_supplementary_table-and-figures.pdf]

Supplementary Data

Foliar application of zinc sulfate and zinc EDTA to wheat leaves: differences in mobility, distribution and speciation

Doolette, C.L.*^, Read, T.L.*, Li. C., Scheckel, K.G., Donner, E., Kopittke, P.M., Schjoerring, J.K., Lombi, E.

*contributed equally to this work
^corresponding author

Supplementary protocol S1:

Preparation of XAS standards

Supplementary data:

XFM analysis of transverse leaf cross section

XFM analysis of a leaf treated with ZnEDTA and corresponding transects

Supplementary table:

Table S1. Distribution of Zn species in wheat leaves

Supplementary figures:

Fig S1. X-ray fluorescence microscopy (XFM) image of an untreated leaf of wheat

Fig S2. Transverse cross-section of a wheat leaf showing the internalisation of Zn. Light microscope images (top) of ZnEDTA and $ZnSO_4$ treated leaves.

Fig. S3. X-ray fluorescence microscopy (XFM) images of wheat leaves showing the distribution of Zn 3,12 and 24 h after the foliar application of $ZnSO_4$ and ZnEDTA .

Fig. S4. Distribution of Zn in wheat leaves 3, 12, and 24 h after the foliar application of $ZnSO_4$ (A) and ZnEDTA (B) from XFM data.

Fig S5. XFM image of a leaf treated with a droplet of ZnEDTA (1000 mg Zn L^{-1}) and removed after 3 h.

Fig. S6. Zinc Kα-edge XANES spectra of standard compounds used in the linear combination fitting of sample spectra.

Fig. S7. Zinc K_{α}-edge XANES spectra showing the difference between the spectra for Zn in the leaf tissues at the site of fertiliser application following exposure to $ZnSO_4$ or ZnEDTA.

Fig. S8. Zinc K_{α}-edge XANES spectra showing the difference between speciation at the site of fertiliser application (blue, 100%) and at a location on the leaf where the maximum signal intensity is 10% of that at the site of application (red, 10% L).

Fig. S9. The background distribution of Zn species in $ZnSO_4$ and ZnEDTA treated leaves as determined from LCF of the K_{α}-edge XANES spectra. Standard errors for percentage contributions are shown in Table S1.

Preparation of XAS standards

Zinc-citrate

Zinc-citrate was prepared by adding 0.2 mL of a $ZnSO_4$ solution (1000 mg Zn/L) to 0.2 mL of a citrate solution (20 mM). Fifty µL of MES buffer (0.05 mL) and 150 mg of glycerol (0.120 mL of a 100% solution) were then added to the solution. The nominal Zn concentration was 350 mg Zn/L.

Zinc-phytate

Zinc-phytate was prepared by adding 0.2 mL of a $ZnSO_4$ (1000 mg Zn/L) solution to 0.2 mL of a phytate solution (20 mM) purchased from Sigma Aldrich (Sigma P-8810). Fifty µL of MES buffer and 150 mg of glycerol (0.120 mL of a 100% solution) were then added to the solution. The nominal Zn concentration was 350 mg Zn/L.

Zinc-cysteine

Zinc-cysteine was prepared by adding 0.2 mL of a $ZnSO_4$ solution (1000 mg Zn/L) to 0.2 mL of a cysteine solution (20 mM). Fifty µL of MES buffer (0.05 mL) and 150 mg of glycerol (0.120 mL of a 100% solution) were then added to the solution. The nominal Zn concentration was 350 mg Zn/L.

Zinc-polygalacturonate

Zinc-polygalacturonate was prepared by adding 0.2 mL of a $ZnSO_4$ solution (500 mg Zn/L) to 0.2 mL of a polygalacturonate solution (5% w/v). Fifty µL of MES buffer and 150 mg of glycerol (0.120 mL of a 100% solution) were then added to the solution. The nominal Zn concentration was 175 mg Zn/L.

Zinc-histidine

Zinc-histidine was prepared by adding 0.2 mL of a $ZnSO_4$ solution (1000 mg Zn/L) to 0.2 mL of a histidine solution (20 mM). Fifty µL of MES buffer (0.05 mL) and 150 mg of glycerol (0.120 mL of a 100% solution) were then added to the solution. The nominal Zn concentration was 350 mg Zn/L.

Zinc-sulfate

Zinc-sulfate was prepared as a 500 mg Zn/L solution in ultrapure deionised water. Glycerol (150 mg, 100% solution) was added to 0.4 mL of the solution prior to analysis.

Zinc-EDTA

Zinc-EDTA was prepared by adding 0.2 mL of a $ZnSO_4$ solution (1000 mg Zn/L) to 0.2 mL of an EDTA solution (4470 mg EDTA/L), giving a molar ratio of Zn:EDTA of 1:1 in the final solution and a nominal Zn concentration of 500 mg Zn/L.

XFM analysis of transverse leaf cross section

X-ray fluorescence microscopy (XFM) analysis of a leaf cross section was performed to demonstrate the penetration of Zn into leaf tissue following foliar application of Zn as ZnEDTA and $ZnSO_4$ to wheat (*Triticum aestivum* cv Shield) leaves. One 5 µL droplet of ZnEDTA or $ZnSO_4$ (1000 mg Zn L^{-1}, 0.05% Tween20) was applied to the adaxial side of a wheat leaf, which was placed in a petri dish with slits in the side and moist filter paper, to prevent the droplet from drying.. The droplet was removed after 3 h by sequentially rinsing with 2% nitric acid (HNO_3), 3% ethanol and deionised water and wiping with a KimTech wipes as described in the main text. The leaves were then sectioned into 2 cm lengths (with the droplet in the centre of the section) and mounted in agar. Thin transverse cross-sections were then collected through the centre of the droplet application site using a Teflon-coated razor blade. Leaf sections were then scanned at the Australian Synchrotron XFM beamline.

Briefly, single energy X-rays were selected using a Si(111) monochromator, and two Kirkpatrick-Baez mirrors were used to form a 2 × 2 μm^2 focus on the sample (Paterson et al., 2011). X-ray fluorescence emitted by the sample (and subsequent elemental maps) were collected using an incident energy of 12,900 eV and a 384-element Maia revision D detector, in backscatter geometry. The sample leaf was analysed continuously horizontally with a sampling interval of 2 µm and a vertical step size of 2 µm. To avoid damaging the sample during XFM scanning, the transit time for each pixel was 2 ms (Lombi *et al*., 2011). The X-ray fluorescence (XRF) spectra were analysed using GeoPIXE (Ryan, 2000; Ryan and Jamieson, 1993).

Results are shown in Figure S2.

XFM analysis of a leaf treated with ZnEDTA and corresponding transects

We were unable to collect data for ZnEDTA treated leaves from the Advanced Photon Source (APS) as was collected for $ZnSO_4$ treated leaves in Experiment 2. However, the same data analysis (Figure S5) was performed on data collected from the scan of a ZnEDTA treated leaf collected in Experiment 1 from the Australian Synchrotron XFM beamline.

Results are shown in Figure S5.

Supplementary table

Table S1. Proportion of Zn species in wheat leaves treated with foliar application of ZnEDTA and $ZnSO_4$ and the percentage variation in the calculated values shown in brackets. PG: polygalacturonic acid. Three replicates are shown for background Zn.

	Zn-citrate	Zn-PG	Zn-phytate	Zn-cysteine	Zn-phosphate	ZnEDTA	Zn-histidine	*R*-factor
ZnEDTA								
10% R		20 (8)	48 (4)	34 (4)		9 (7)		0.0069
25% R		9 (7)	45 (3)	28 (3)		29 (6)		0.0042
50% R			46 (2)	4 (2)		60 (1)		0.0023
100% Max.			42 (1)			66 (1)		0.0019
50% L			39 (2)	3 (2)		67 (1)		0.0022
25% L			45 (3)	10 (3)		55 (2)		0.0046
10%L		11 (10)	50 (4)	21 (4)		30 (9)		0.0079
$ZnSO_4$								
10% R	40 (7)	22 (10)	40 (2)	4 (2)				0.0023
25% R	37 (4)	10 (5)	58 (1)					0.0008
50% R	32 (3)	16 (4)	55 (2)		3 (1)			0.0005
100% Max.		29 (1)	41 (1)	14 (1)	21 (1)			0.0002
50% L	23 (4)	8 (6)	64 (2)	10 (1)				0.0009
25% L	42 (5)	13 (6)	50 (2)					0.0015
10%L	34 (7)	26 (10)	37 (3)	9 (2)				0.0028
Background								
ZnEDTA			28 (4)	82 (4)				0.0147
ZnEDTA			30 (3)	78 (3)				0.0093
ZnEDTA			32 (3)	75 (4)				0.0111
$ZnSO_4$		4 (6)	29 (9)	73 (16)			4 (17)	0.0507
$ZnSO_4$		7 (5)	12 (7)	86 (14)			6 (14)	0.0345
$ZnSO_4$			32 (7)	78 (7)				0.0508

Supplementary figures

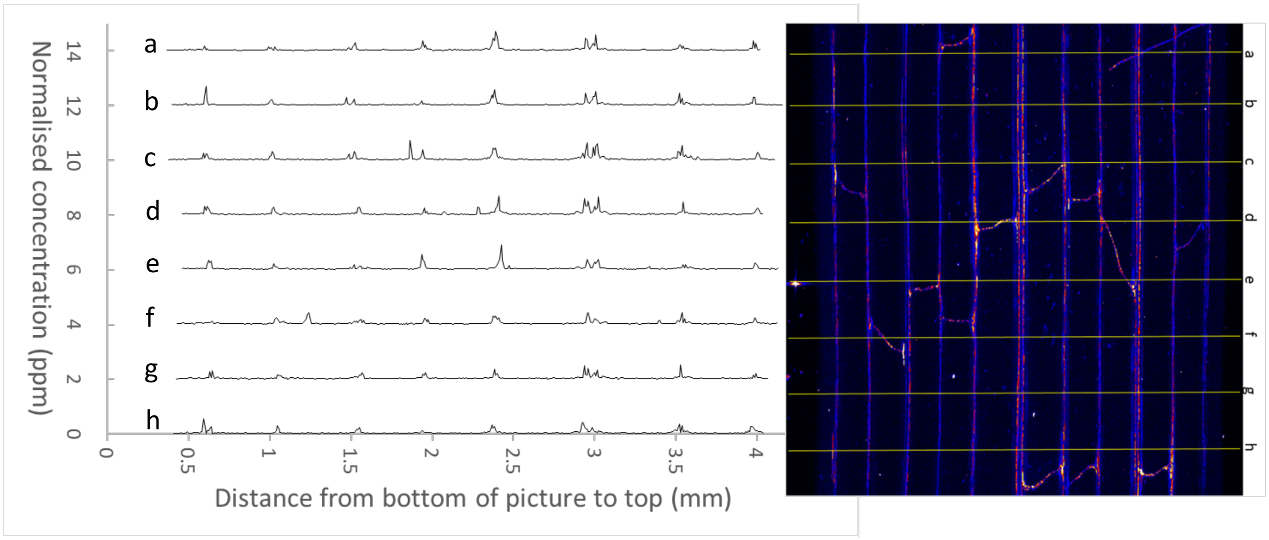

Fig. S1. X-ray fluorescence microscopy (XFM) image of an untreated leaf of wheat showing the presence of Zn in the veins of the leaf, but not in the interveinal areas.

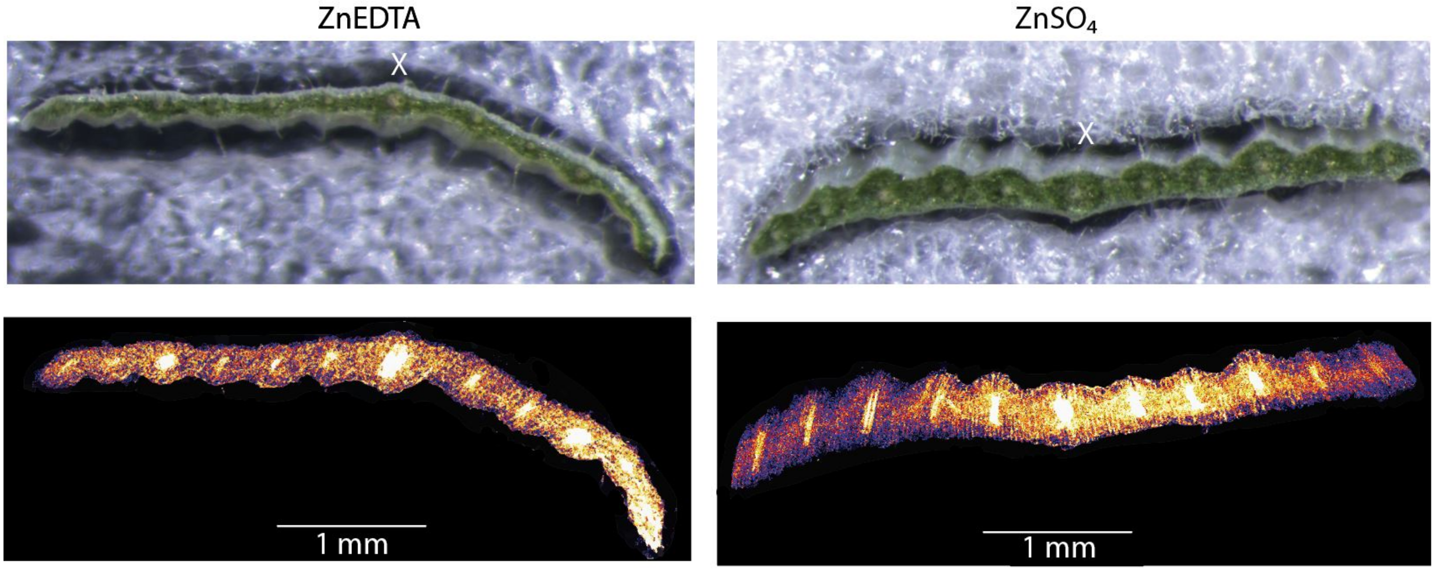

Fig S2. Transverse cross-section of a wheat leaf showing the internalisation of Zn. Light microscope images (top) of ZnEDTA and $ZnSO_4$ treated leaves and the corresponding XFM Zn maps are shown (bottom). The site of droplet (~2.5 mm) application is indicated by the 'X' in the light microscope images (top).

In the $ZnSO_4$ treated leaf, there is a low intensity of Zn on the surface of the leaf where the droplet was applied. However, there is an area of higher intensity underneath the droplet application site indicating internalised Zn. The main veins can be seen as brighter spots along the width of the leaf.

In the ZnEDTA treated leaf, the intensity appears more homogenous throughout the leaf. The homogenous distribution of Zn from the top of the leaf to the bottom, indicates that Zn applied from the surface has been internalised.

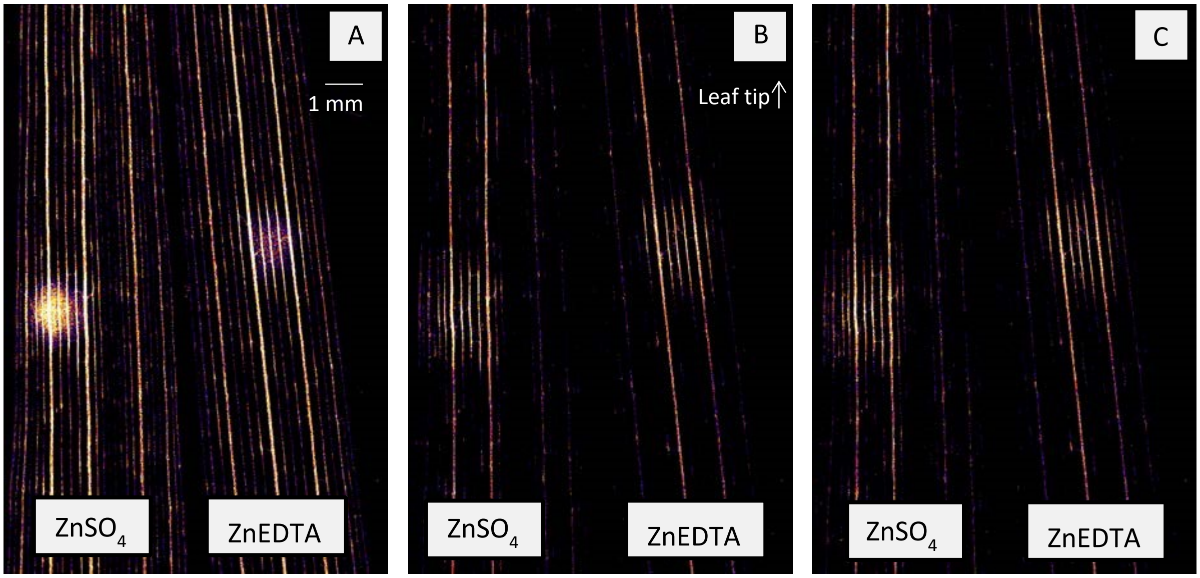

Fig. S3. X-ray fluorescence microscopy (XFM) images of wheat leaves showing the distribution of Zn following foliar application of $ZnSO_4$ and ZnEDTA. The Zn-containing droplets were applied to the leaf surface for 3 h before being removed, with images collected after 3 h (i.e. immediately after removing the droplets) (A), after 12 h (i.e. 9 h after removal) (B) and 24 h (i.e. 21 h after removal) (C). The total size of the area scanned in each image is 13.5 mm × 20 mm.

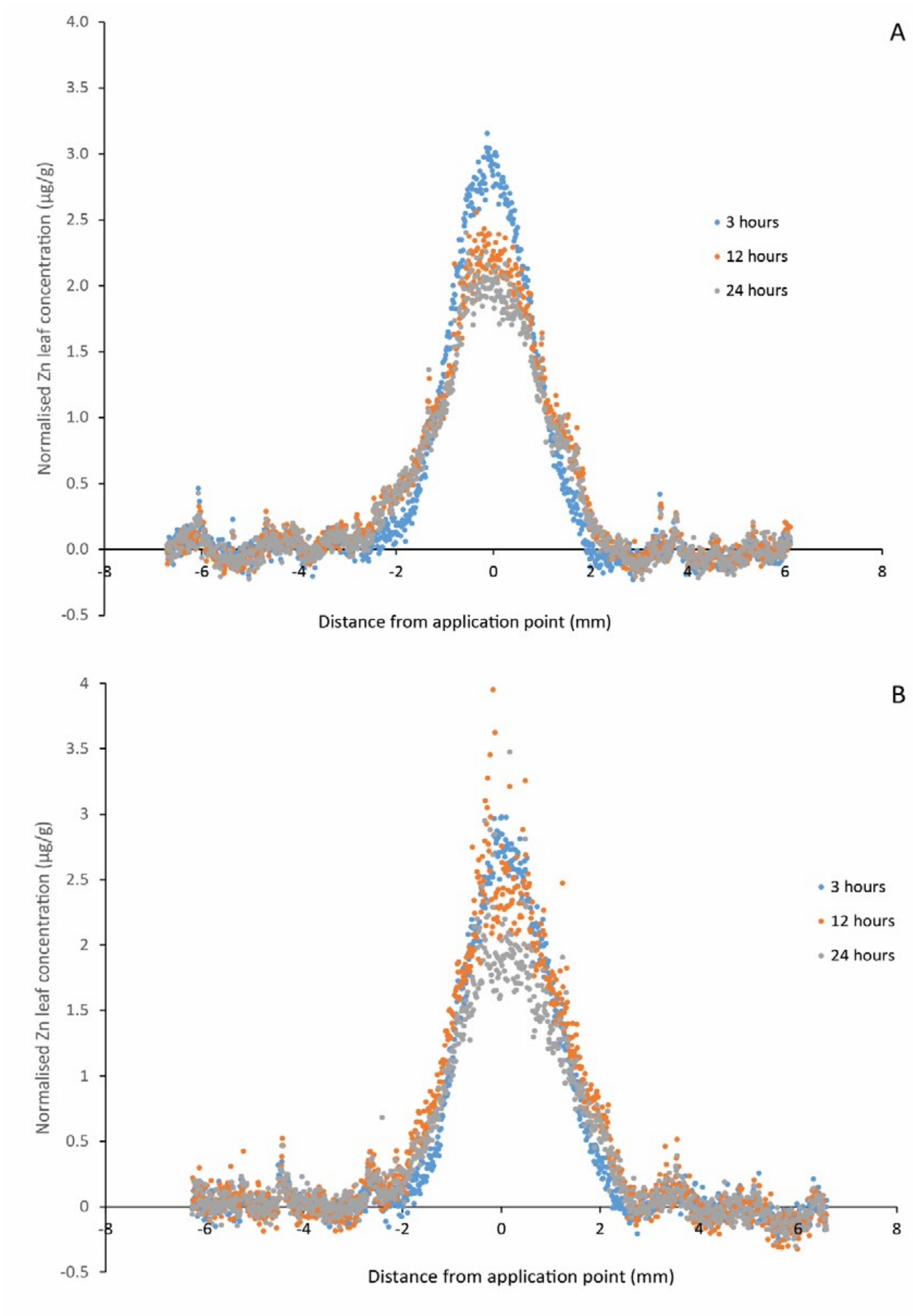

Fig. S4. Distribution of Zn in wheat leaves 3, 12, and 24 h after the foliar application of $ZnSO_4$ (A) and ZnEDTA (B) from XFM data. XFM data were normalised to the background Zn concentrations in leaves.

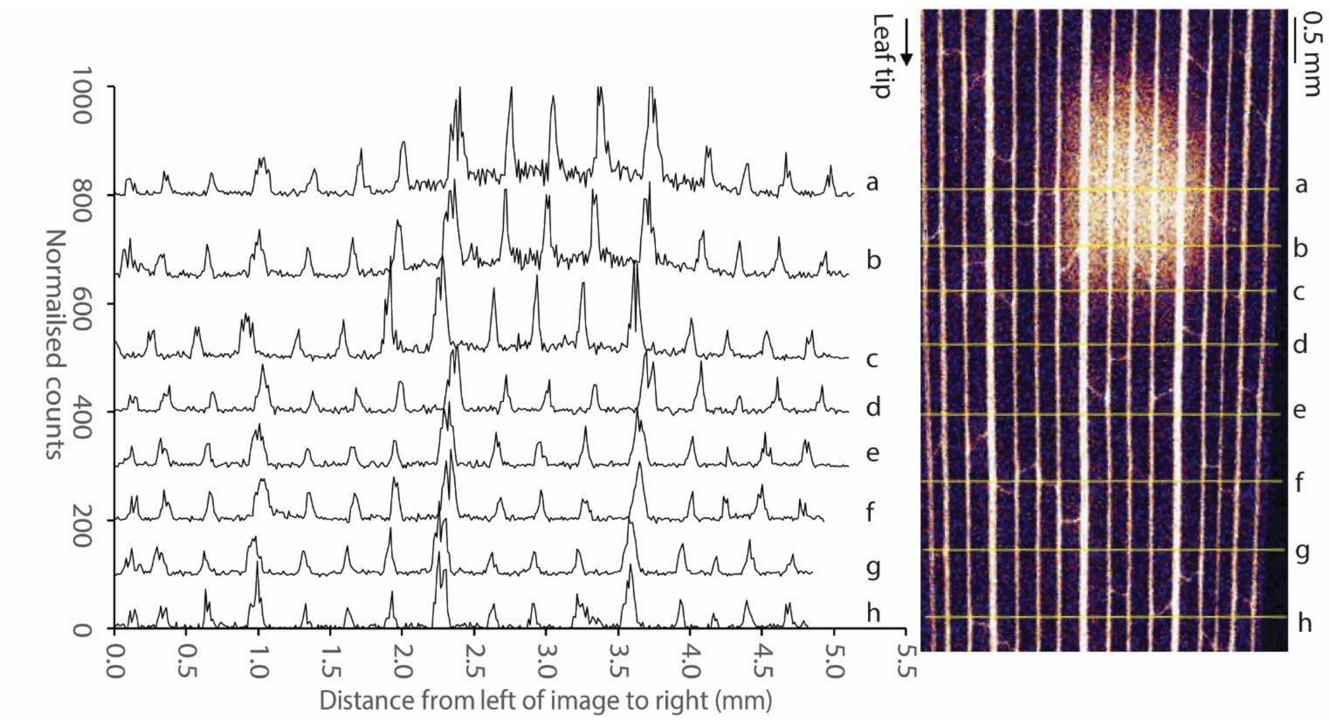

Fig S5. XFM image of a leaf treated with a droplet of ZnEDTA (1000 mg Zn L^{-1}) and removed after 3 h. Line scans were collected across the leaf at locations shown by the yellow lines in the XFM image (transects a–h).

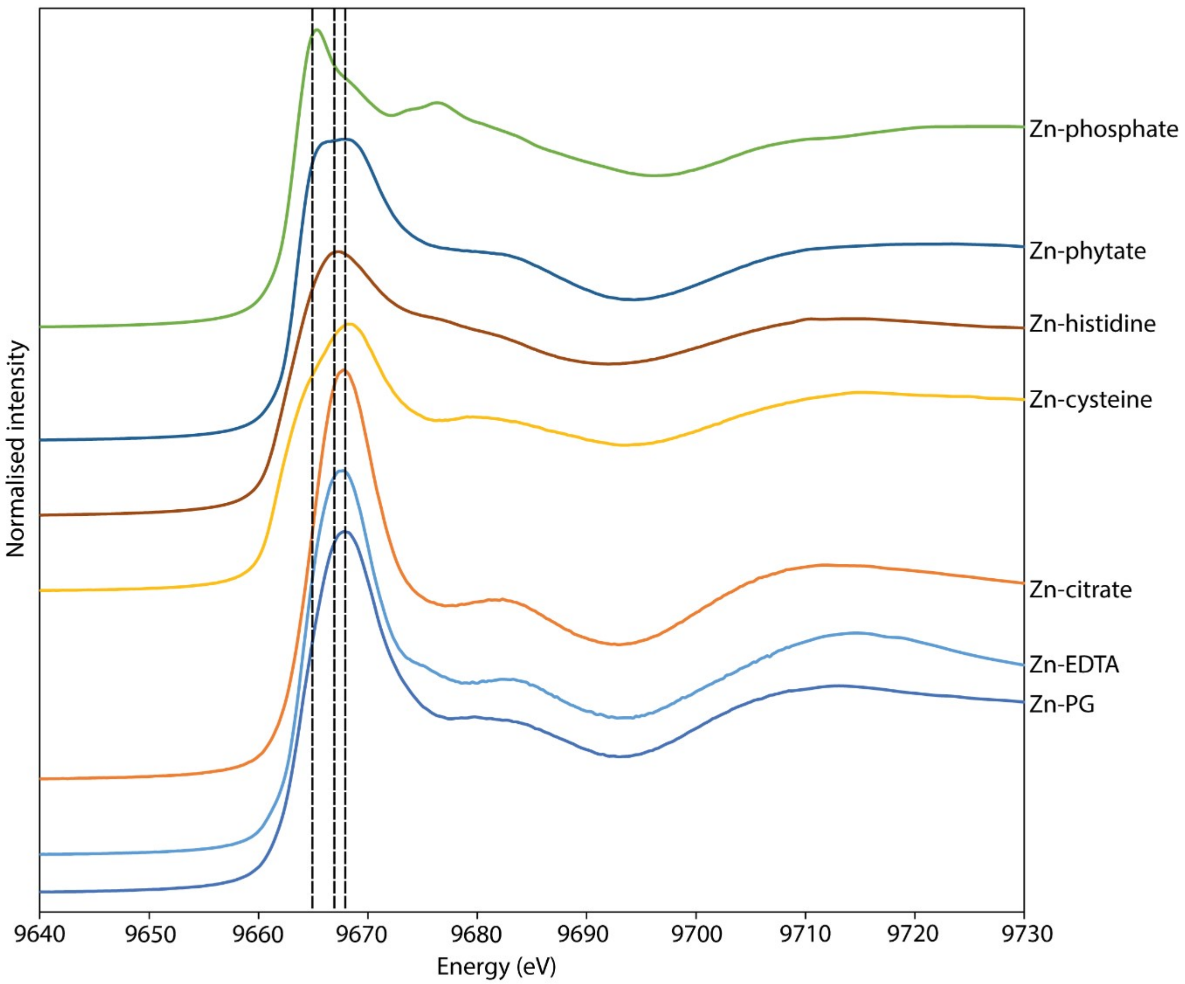

Fig. S6. Zinc Kα-edge XANES spectra of standard compounds used in the linear combination fitting of sample spectra. Zn-PG = Zinc-polygalacturonic acid. The dashed lines are to guide the eye and correspond to energies of 9,665 eV, 9,667 eV and 9,668 eV.

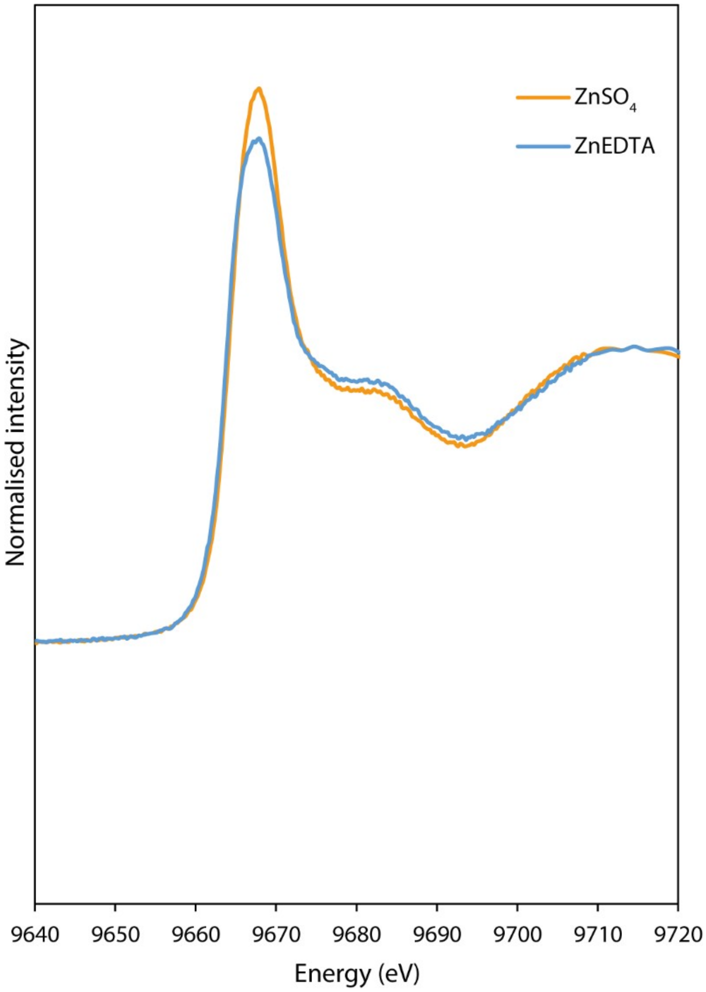

Fig. S7. Zinc K_{α}-edge XANES spectra showing the difference between the spectra for Zn in the leaf tissues at the site of fertiliser application following exposure to $ZnSO_4$ or ZnEDTA.

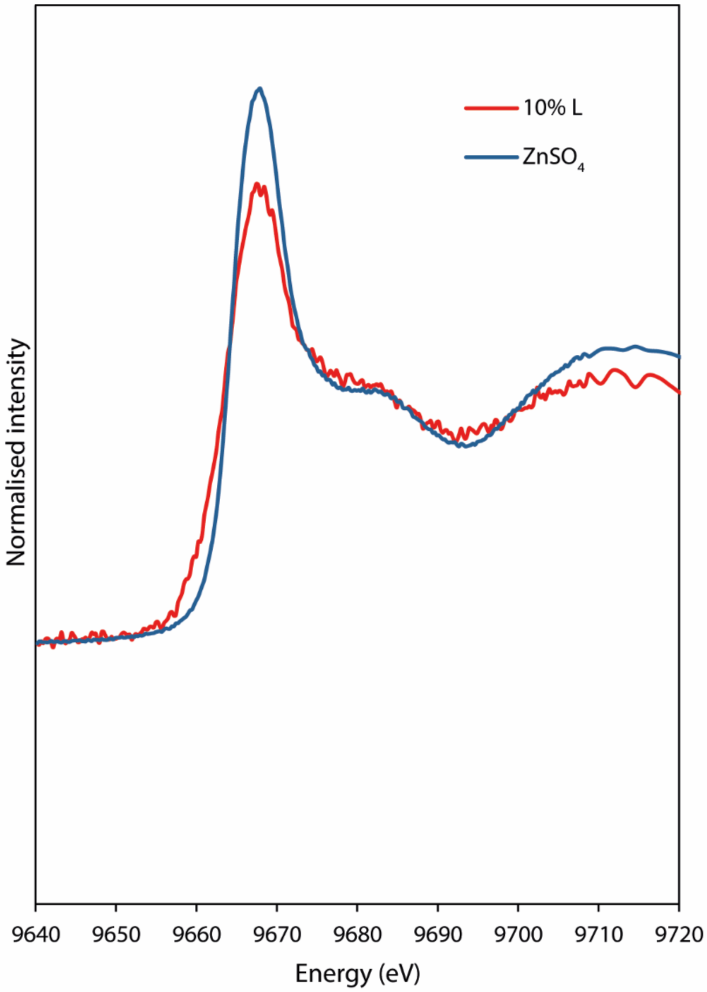

Fig. S8. Zinc K_{α}-edge XANES spectra showing the difference between speciation at the site of fertiliser application (blue, 100%) and at a location on the leaf where the maximum signal intensity is 10% of that at the site of application (red, 10% L).

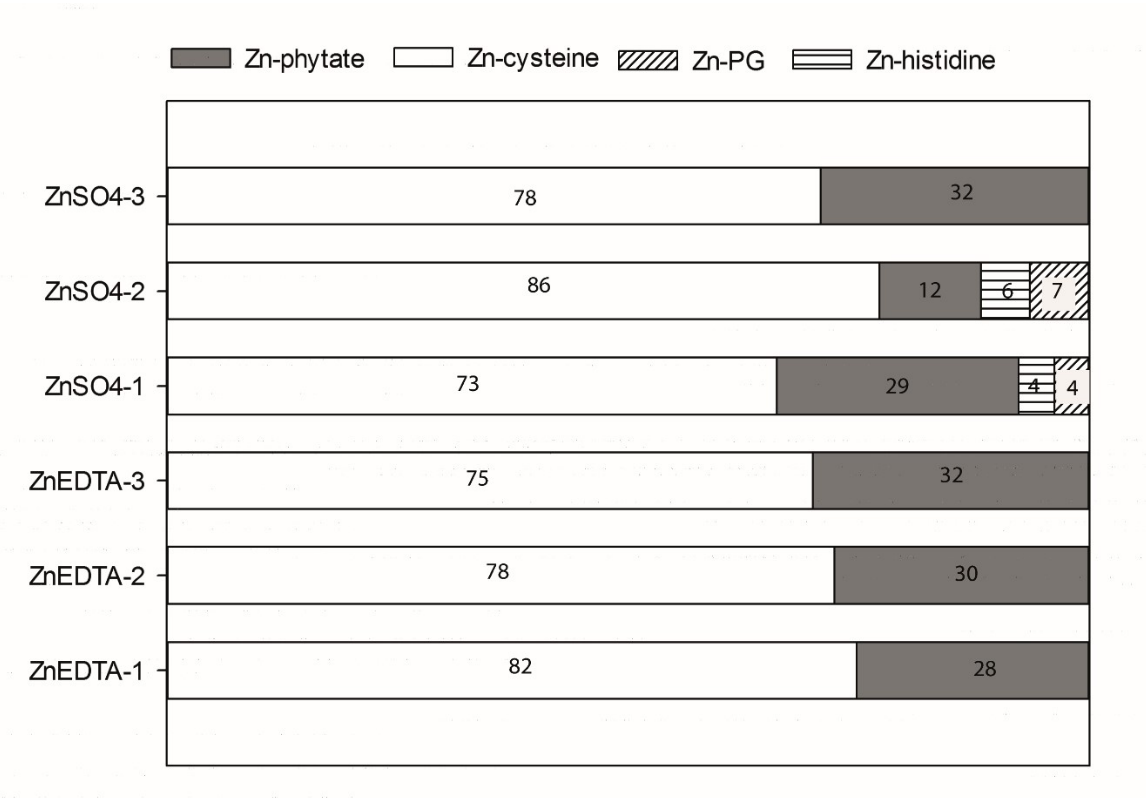

Fig. S9. The background distribution of Zn species in $ZnSO_4$ and ZnEDTA treated leaves as determined from LCF of the K_α-edge XANES spectra. Standard errors for percentage contributions are shown in Table S1.

References

Currie LA. 1968. Limits for qualitative detection and quantitative determination. Application to radiochemistry. Analytical Chemistry **40**, 586-593.

Li H-F, McGrath SP, Zhao F-J. 2008. Selenium uptake, translocation and speciation in wheat supplied with selenate or selenite. New Phytologist **178**, 92-102.

Lombi E, de Jonge MD, Donner E, Kopittke PM, Howard DL, Kirkham R, Ryan CG, Paterson D. 2011. Fast X-Ray fluorescence microtomography of hydrated biological samples. PLOS ONE **6**, e20626.

Paterson D, Jonge MDd, Howard DL, Lewis W, McKinlay J, Starritt A, Kusel M, Ryan CG, Kirkham R, Moorhead G, Siddons DP. 2011. The X-ray fluorescence microscopy beamline at the Australian synchrotron. AIP Conference Proceedings **1365**, 219-222.

Ryan CG. 2000. Quantitative trace element imaging using PIXE and the nuclear microprobe. International Journal of Imaging Systems and Technology **11**, 219-230.

Ryan CG, Jamieson DN. 1993. Dynamic analysis: on-line quantitative PIXE microanalysis and its use in overlap-resolved elemental mapping. Nuclear Instruments and Methods in Physics Research Section B: Beam Interactions with Materials and Atoms **77**, 203-214.
